# A comprehensive study on the oncogenic mutation and molecular pathology in Chinese lung adenocarcinoma patients

**DOI:** 10.1186/s12957-020-01947-z

**Published:** 2020-07-16

**Authors:** Xilin Zhang, Yan Jiang, Huanming Yu, Hui Xia, Xiang Wang

**Affiliations:** 1 Central Laboratory, The First People’s Hospital of Huzhou, No. 158 Guangchang Back Road, Huzhou, 313000 Zhejiang, People’s Republic of China; 2 Department of Cardiothoracic Surgery, The First People’s Hospital of Huzhou, Huzhou, 313000 People’s Republic of China; 3 Department of Pathology, The First People’s Hospital of Huzhou, Huzhou, 313000 People’s Republic of China

**Keywords:** Lung adenocarcinoma, Micropapillary pattern, *EGFR*, *ROS1*, *EML4-ALK*

## Abstract

**Background:**

Several genetic driver alterations have been identified in micropapillary lung adenocarcinoma (MPA). However, the frequency of co-alteration of *ROS1*, *EGFR*, and *EML4-ALK* is yet unclear. Herein, we investigated the relationship between clinicopathologic characteristics and well-identified driver mutations of MPA compared with non-micropapillary lung adenocarcinoma (LA).

**Methods:**

Formalin-fixed paraffin-embedded (FFPE) sections derived from lung adenocarcinoma patients who never received adjuvant chemotherapy or radiation therapy prior to surgical resection were collected from October 2016 to June 2019. *EGFR* mutations, *ROS1* rearrangements, and *EML4-ALK* fusion were identified in a set of 131 MPA and LA cases by using the amplification refractory mutation system (ARMS). The response rate and duration of response were assessed using Response Evaluation Criteria in Solid Tumors version 1.1 (RECIST 1.1).

**Results:**

*EGFR* mutations had occurred in 42 (76.4%) MPA patients and 42 (55.3%) LA patients. Interestingly, *ROS1* rearrangements were highly enriched only in the MPA cases (6/55, 10.9%) but rarely in the LA cases (1/76, 1.3%). Furthermore, 7.3% (4/55) MPA samples had double gene mutations, while only 1.3% (1/76) LA cases had double gene alterations. Of 5 patients with harboring two driver oncogene mutations, four patients (80%) obtained partial response, and one patient (20%) suffered recurrence.

**Conclusions:**

A higher prevalence of *ROS1* rearrangement or combined mutations of *ROS1*, *EGFR*, and *EML4-ALK* may play a critical role in the tumorigenesis of MPA. These findings provide a novel therapeutic strategy for patients with malignant MPA through combining TKIs than one TKI.

## Introduction

Lung cancer remains to be the leading cause of cancer-related death worldwide, and the most frequent histological subtype is lung adenocarcinoma [1]. Lung adenocarcinoma usually includes various histological subtypes, including solid, lepidic, acinar, papillary, and micropapillary patterns [2] according to the International Association for the Study of Lung Cancer (IASLC)/American Thoracic Society (ATS)/European Respiratory Society (ERS) [3, 4]. Numerous studies have reported that lung adenocarcinoma with a micropapillary pattern (MPA) shows more aggressive behaviors and worse survival than other histological subtypes of lung adenocarcinoma (LA) [5–7].

Several oncogenic drivers have been identified in lung adenocarcinoma, including mutations in the epidermal growth factor receptor (EGFR) [8], fusions of anaplastic lymphoma kinase (ALK) [9], and rearrangements of ROS proto-oncogene 1 receptor tyrosine kinase (ROS1) [10]. Accumulating evidence demonstrated that mutations of *EGFR* were identified in 15–30% of lung adenocarcinomas in Caucasians [11] and 40–60% in East Asians [12–14], indicating that the frequency of activated mutations of *EGFR* depends on ethnicity. Besides, *ALK* fusions were firstly identified in 2007 and occurred in approximately 3–7% of all lung adenocarcinoma patients, and the most common form was echinoderm microtubule-associated protein-like 4/anaplastic lymphoma kinase (*EML4-ALK*) [15]. In the same year, an additional novel oncogenic fusion gene, *ROS1*, was identified, which accounted for 1–2% of all lung adenocarcinoma patients. Of special interest, this ratio increased to 5–7% for lung adenocarcinoma patients without *EGFR*/*KRAS*/*BRAF*/*ALK* mutations [16]. With the development of tyrosine kinase inhibitors (TKIs), TKIs served as the first-line option for patients harboring *EGFR*-sensitive mutations, *ALK* fusions, or *ROS1* rearrangements. Therefore, the discovery of TKIs against *EGFR* gene activation mutations (for example, gefitinib and erlotinib) [17] and *ALK* or *ROS1* gene rearrangements (for example, crizotinib) [18, 19] has significantly improved the outcomes of patients. For detection of *ROS1* and *EML4-ALK* fusions, immunohistochemistry (IHC), next-generation sequencing (NGS), ARMS-polymerase chain reaction (ARMS-PCR), and fluorescence in situ hybridization (FISH) have been widely used [20]. Although FISH is the gold standard test, it is expensive and time-consuming. By contrast, ARMS-PCR is a more sensitive and feasible approach compared to FISH and IHC [21].

There is growing evidence that *EGFR* gene mutations are more common in MPA than in LA, while *ROS1* gene rearrangement has not been clearly demonstrated in MPA patients [22–24]. Moreover, the co-existence of *EGFR* gene mutations, *ALK* gene fusions, and *ROS1* gene rearrangements has been reported in a few lung adenocarcinoma cases [25–27], but the co-alteration of *ROS1*, *EGFR*, and *EML4-ALK* in MPA remains unclear.

The molecular features of MPA may differ from other histopathological subtypes of lung adenocarcinoma [28]; however, the determinate information is not available. In the present study, we investigated the relationship between the most common driver mutations and the pathology features in Chinese lung adenocarcinoma patients.

## Materials and methods

### Patient selection

A total of 131 lung adenocarcinoma patients were enrolled in the First People’ Hospital of Huzhou from January 2016 to June 2019. Of them, 55 cases harbored at least 5% micropapillary component [22], who were represented as MPA, and the remaining cases (43 solid, 20 acinar, and 13 lepidic) were defined as LA. All of them were initially diagnosed with lung adenocarcinoma and had not received neoadjuvant or adjuvant chemotherapy or radiation therapy prior to surgical resection. The pathological diagnosis was confirmed and classified using hematoxylin and eosin staining by two certified pathologists (Qilin Shi and Xiaolan Zhang from the First People’s Hospital of Huzhou) based on the IASLC/ATS/ERS multidisciplinary classification system [3]. All specimens contained 60% of tumor cells and sufficient tissues for further mutational analysis. Clinical information collected includes age, gender, tumor differentiation, tumor size, smoking history, lymphatic invasion, pleural invasion, tumor node metastasis (TNM) stage, micropapillary pattern, and prognostic data. This study was undertaken with the agreement of our hospital ethics committee, and the informed consent signature was provided by all patients.

### Evaluations of treatment

After surgical resection, 5 patients were treated with 3 months chemotherapy following with targeted therapy (TKIs). Tumors were evaluated during the treatment with chemotherapy, *EGFR*-TKIs or *ROS1*/*EML4-ALK* inhibitors every 6 weeks. Efficacy was obtained using CT scan according to the Response Evaluation Criteria in Solid Tumors version 1.1(RECIST 1.1) [29]. Objective response rate (ORR) included complete response (CR), partial response (PR), stable disease (SD), and progressive disease (PD).

### Mutational analysis

For *EGFR* mutation analysis, DNA from FFPE tissue sections was extracted by using a QIAamp DNA FFPE tissue kit (cat. No. 56404, Qiagen, Germany) according to the manufacturer’s instructions. *EGFR* mutations within exons 18–21 were identified with commercial kits (Human EGFR mutation Detection Kit, cat. No. YZYMT-002-A, YZY Medical Co., Ltd., Wuhan, China) using ARMS. It was capable of detecting the following mutations: three in exon 18 (G719S, G719C, and G719A), one deletion in exon 19, two mutations in exon 20 (T790M and S768I), three insertions in exon 20, and two mutations in exon 21 (L858R and L861Q). Briefly, ARMS-PCR amplification (37 °C for 10 min; 95 °C for 5 min; 40 cycles of 95 °C for 15 s, and 60 °C for 60 s) was performed using the 7500 system (Applied Biosystems; Thermo Fisher Scientific, Inc.). For *EML4-ALK* and *ROS1* fusion analysis, RNA was isolated from FFPE sections by applying a RNeasy FFPE tissue kit (cat. No. 73504, Qiagen, Germany) according to the standard procedure. Complementary DNA was reverse-transcribed using the PrimeScript RT reagent kit (Takara Biotechnology Co., Ltd.), according to the manufacturer’s protocol. RNA (500 ng), 2 μl PrimeScript^TM^ RT Master Mix (Perfect Real Time) (Takara Biotechnology Co., Ltd.), and RNase free water (up to 10 μl) were mixed together and incubated at 37 °C for 15 min and 85 °C for 5 s. Then, ARMS-PCR amplification (37 °C for 10 min, 95 °C for 5 min, 40 cycles of 95 °C for 15 s, and 60 °C for 35 s) was performed using the 7500 system (Applied Biosystems; Thermo Fisher Scientific, Inc.). *ROS1* fusions (fusion partners for *ROS1*: *CD74*, *SLC34A2*, *SDC4*, *EZR*, *LRIG3*, *TPM3*, and *GOPC*) and *EML4-ALK* rearrangement were identified with commercial kits (Human ROS1 fusion Detection Kit, cat. No. YZYMT-022 and Human EML4-ALK fusion Detection Kit, cat. No. YZYMT-021, YZY Medical Co., Ltd., Wuhan, China).

### Statistical analysis

Comparisons between two or more categorical variables were conducted using the Chi-square test and Fisher’s exact test. Data were statistically performed on *SPSS22.0* (Chicago, IL, USA). The results were considered statistically significant at *p* < 0.05.

## Results

### Clinicopathologic characteristics

**Fig. 1:**
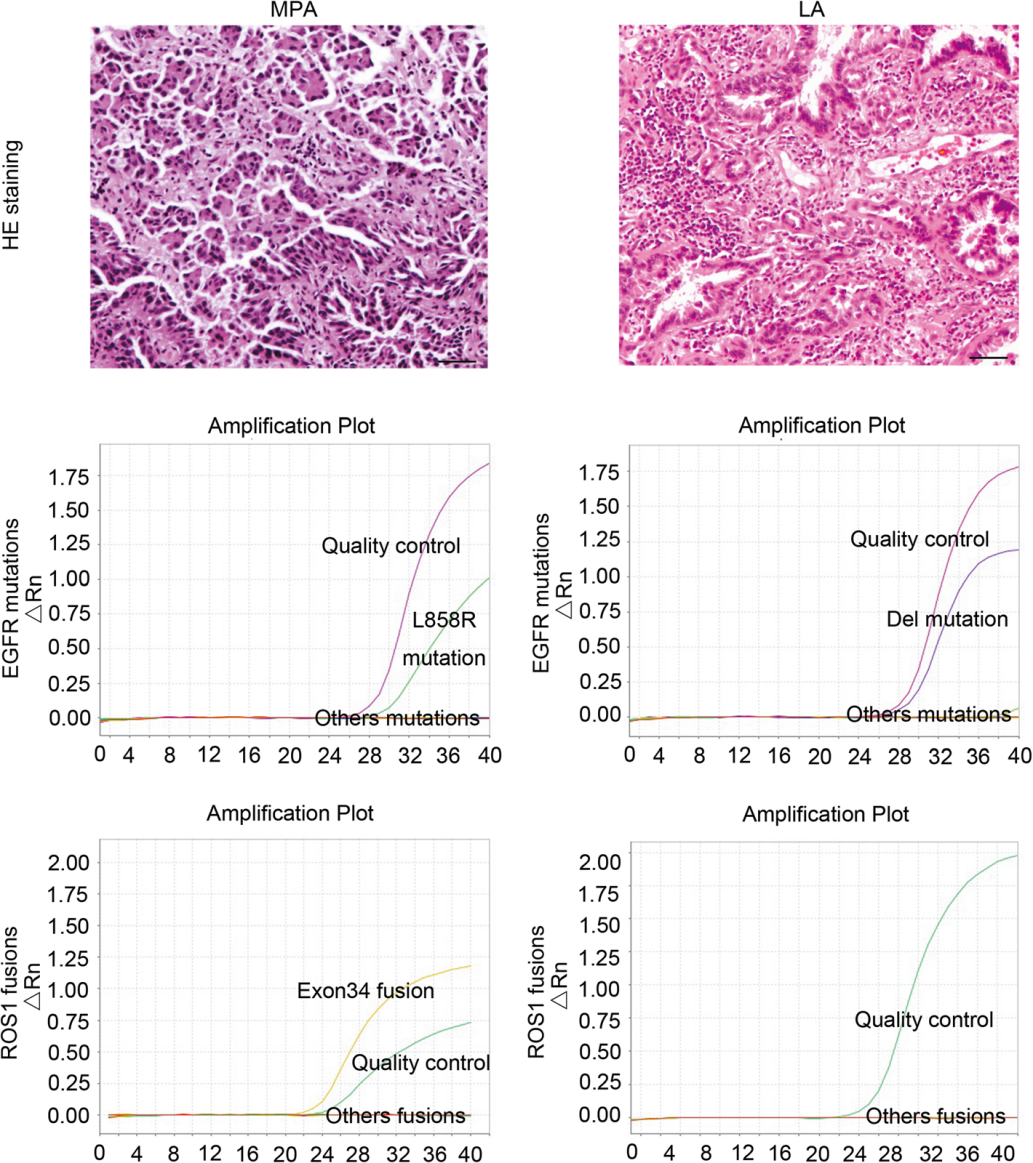
Immunohistochemical staining and mutation analysis of MPA and LA patients. Three micrometers FFPE sections of MPA (patient 1) and LA (patient 16) were immunostained with hematoxylin and eosin (× 100 magnification). Scale bar 20 μm. Abbreviations: MPA, micropapillary lung adenocarcinoma; LA, lung adenocarcinoma without micropapillary component

Among MPA cases, 36 patients fell into stage I, 11 into stage II, 6 into stage III, and 2 into stage IV. In LA, 39 (16 solid, 16 acinar, and 7 lepidic) fell into stage I, 12 (10 solid, 1 acinar, and 1 lepidic) into stage II, 8 (4 solid, 1 acinar, and 3 lepidic) into stage III, and 17 (13 solid and 4 lepidic) into stage IV. Seventy-two people had no smoking history, and fifty-nine were smokers, including former smokers and current smokers. The histological images of MPA and LA are presented in Fig. 1.

The MPA group consisted of 24 (43.6%) women and 31 (56.4%) men with an age at diagnosis ranging from 31 to 82 years (mean 62). In comparison, the LA group was composed of 38 (50.0%) women and 38 men (50.0%) with a mean age of 65 years (range, 46–81 years). Compared to LA, MPA patients had a significantly younger age at diagnosis (*p* = 0.008), positive lymph node metastasis (*p* = 0.03), positive pleural invasion (*p* = 0.036), and earlier disease staging (*p* = 0.024) (Table 1). However, the micropapillary pattern was not significantly associated with gender, smoking history, tumor size, and tumor differentiation (Table 1).

**Table 1:** Clinicopathologic and molecular characteristics of MPA and LA cases

Variables	MPA (***n*** = 55)	LA(***n*** = 76)	χ^2^	***p***
Gender			0.518	0.485
Female	24(43.6%)	38(50.0%)		
Male	31(56.4%)	38(50.0%)		
Age(years)			7.452	0.008**
> 65	20(36.4%)	46(60.5%)		
≤ 65	35(63.6%)	30(39.5%)		
Smoking history			0.191	0.723
Ever	26(47.3%)	33(43.4%)		
Never	29(52.7%)	43(56.6%)		
Tumor size(cm)			0.058	0.851
> 3.0	17(30.9%)	25(32.9%)		
≤ 3.0	38(69.1%)	51(67.1%)		
Lymphovascular invasion			1.645	0.225
Present	17(30.9%)	16(21.1%)		
Absent	38(69.1%)	60(78.9%)		
Tumor differentiation			0.179	0.835
Well/moderate	43(78.2%)	57(75.0%)		
Poor	12(21.8%)	19(25.0%)		
N status			4.291	0.03*
N0	31(56.4%)	56(73.7%)		
N1/N2	24(43.6%)	20(26.3%)		
Pleural invasion			4.117	0.036*
Present	15(27.3%)	10(13.2%)		
Absent	40(72.7%)	66(86.8%)		
Stage			5.701	0.024*
I/II	47(85.5%)	51(67.1%)		
III/IV	8(14.5%)	25(32.9%)		
Mutation status				
*EGFR* mutation	42(76.4%)	42(55.3%)	6.175	0.016*
*EML4-ALK* fusion	3(5.5%)	1(1.3%)	1.846	0.309
*ROS1* fusion	6(10.9%)	1(1.3%)	5.806	0.041*

*MPA* micropapillary lung adenocarcinoma, *LA* lung adenocarcinoma without micropapillary component

*p<0.05; **p<0.01 compared with LA

### Mutational status of classic oncogenes

MPA and LA groups included 42 (76.4%) and 42 (55.3%) *EGFR* mutations, respectively. Interestingly, *ROS1* rearrangements were highly enriched only in the MPA group (6/55) but rarely in the LA group (1/76) (*p* = 0.041, Table 1). Furthermore, we also discovered that different genetic driver alterations often co-existed in the MPA group, for instance, *EGFR* combined with *ROS1* (*n* = 2) and *EML4-ALK* combined with *ROS1* (*n* = 2), while only one LA case harbored *EGFR* combined with *ROS1*, suggesting that co-existent alterations of *EGFR*, *ROS1*, and *EML4-ALK* were more frequent in MPA than in LA (*p* = 0.043, Table 2). These results indicate the potential combined treatments of MPA with two different TKIs targeted to *EGFR* and *ROS1*.

**Table 2:** Coexistent genetic alterations including *EGFR*, *ROS1*, and *EML4-ALK* in MPA and LA cases

Variables	MPA(*n* = 55)	LA(*n* = 76)	*p*
Single alteration			0.028*
EGFR +	40(72.7%)	41(53.9%)	
ROS1 +	2(3.6%)	0	
EML4-ALK+	1(1.8%)	1(1.3%)	
Double alteration			0.043*
EGFR+; ROS1+	2(3.6%)	1(1.3%)	
ROS1+; EML4-ALK+	2(3.6%)	0	
No alteration			0.001**
EGFR-; ROS1-; EML4-ALK-	8(14.5%)	33(43.5%)	

*MPA* micropapillary lung adenocarcinoma, *LA* lung adenocarcinoma without micropapillary component, *EGFR+* EGFR mutation, *EGFR-* EGFR wild type, *ROS1+* ROS1 fusion, *ROS1-* no ROS1 fusion, *EML4-ALK+* EML4-ALK fusion, *EML4-ALK-* no EML4-ALK fusion

*p<0.05; **p<0.01 compared with LA

To further explore the association between clinicopathologic characteristics and genetic driver alterations, we analyzed the general information and therapeutic outcomes in 5 lung adenocarcinoma patients with combined mutations. After surgical resection, all patients were treated with 3 months chemotherapy following with targeted therapy (TKIs). Follow-up data were accessible to all 5 patients after postoperative ranging from 0 to 12 months (median 10.8 months). All patients survived to the last day of follow-up. The results showed that four patients partially responded, and one patient suffered a recurrence according to RECIST 1.1 [29] (Table 3).

**Table 3:** General information and therapeutic outcomes on 5 patients with coexistent genetic alterations including *EGFR*, *ROS1*, and *EML4-ALK* in MPA and LA patients

Patients	1	2	3	4	5
Gender	Female	Male	Female	Male	Female
Age (years)	52	31	45	71	75
Smoking history	Never	Never	Never	Ever	Never
Lymphovascular invasion	Absent	Present	Absent	Absent	Absent
Tumor differentiation	Moderate	Moderate	Moderate	Moderate	Moderate
Stage	T1bN0M0	T1cN1M0	T1cN0M0	T1bN0M0	T1cN0M0
Pathological type	MPA	MPA	MPA	MPA	LA
Mutation status	ROS1+; EML4-ALK+	ROS1+; EML4-ALK+	EGFR L858R+; ROS1+	EGFR L858R+; ROS1+	EGFR 19-del+; ROS1+
Therapeutic intervention	Surgery; chemotherapy; crizotinib	Surgery; chemotherapy; crizotinib	Surgery; chemotherapy; EGFR inhibitors	Surgery; chemotherapy; EGFR inhibitors	Surgery; chemotherapy; EGFR inhibitors
Outcomes	Partial response	Partial response	Partial response	Recurrence	Partial response

*MPA* micropapillary lung adenocarcinoma, *LA* lung adenocarcinoma without micropapillary component, *EGFR+* EGFR mutation, *ROS1+* ROS1 fusion, *EML4-ALK+* EML4-ALK fusion

## Discussion and conclusions

Accumulating evidence indicates that the co-existence of classic oncogenes, involving *EGFR*, *ALK*, *ROS1*, and *MET*, was identified in lung adenocarcinoma patients, especially in younger and women patients without a smoking history. However, few studies have focused on the frequency of two-driver alterations of *EGFR*, *ROS1*, or *EML4-ALK* in MPA and LA. Therefore, we investigated the relationship between the most common oncogenic mutations and molecular pathological characteristics in Chinese lung adenocarcinoma patients.

Consistent with previous reports [30, 31], we here discovered that MPA has positive lymph node metastasis, positive pleural invasion, and earlier disease staging compared with LA (Table 1). Increasing studies have shown that a micropapillary component was associated with lymph node metastasis, pleural invasion, and an early recurrence in stage I patients, suggesting MPA had a poorer prognosis compared with those without micropapillary component or other histological subtypes [32–39]. Our results further implied that the higher prevalence of lymph node metastasis and pleural invasion may be a valuable poor prognostic marker for MPA.

An investigation of 15 MPAs revealed that the mutational status of *EGFR*, *KRAS*, and *BRAF* harbored 73% mutually exclusive mutations in the Western population [40]. A study involving 21 micropapillary predominant lung adenocarcinoma patients showed that oncogenic mutations in *EGFR*, *HER2*, and *RET* were apparently frequent in 95.2% Chinese people [22]. Here, our results manifested that the majority (47 out of 55, 85.5%) of MPA harbored the genetic driver alterations of *EGFR* (76.4%), *ROS1* (10.9%), or *EML4-ALK* (5.5%) from a Chinese cohort. Though previous cohort detected no *ROS1* fusions [22], two another independent teams found *ROS1* rearrangements in MPA [23, 24]. Therefore, there are no consistent conclusions about *ROS1* rearrangements in MPA patients. Here, our cohort reported that 6 MPA cases possessed *ROS1* rearrangements. In the past, oncogenic mutations involving *EGFR*, *KRAS*, *ALK*, *RET*, *ROS1*, and *MET* were regarded as mutually independent events. However, two or more cancer-associated gene alterations were recently found in lung adenocarcinoma cases [41–44]. Our study indicated that 3.8% of lung adenocarcinoma cases harbored two-driver alterations of *EGFR*, *ROS1*, or *EML4-ALK*, including 7.3% of MPA cases and 1.3% of LA cases, and this result was in agreement with recent reports [41–45].

According to previous reports, patients with co-alterations of *EGFR*, *ALK*, *ROS1*, and other oncogenic drivers showed distinctive clinical responses to TKIs in lung adenocarcinoma [44, 46–48]. Yang et al. demonstrated that the median progression-free survival of gefitinib was 11.2 months in patients with concomitant *EGFR* and *ALK* alteration [49]. Mao et al. indicated that the median progression-free survival of *EGFR*-TKIs and/or *ALK/ROS1* inhibitor was 6.6 months in patients with concomitant *EGFR* and *ALK* alteration [44]. However, 75% of patients with crizotinib treatment obtained disease control [44]. In the present study, all patients undertook the operation and chemotherapy initially and undertook subsequently targeted therapy. In addition, Watanabe et al. showed that the micropapillary component was associated with an early recurrence in stage I patients but not in advanced-stage patients, indicating MPA retained a high risk of early recurrence after 1-year surgery [39]. In the present study, among five patients with concomitant alterations of *EGFR*, *ROS1*, and *EML4-ALK*, four patients partially responded and one patient suffered a recurrence during 1 year follow-up. Our study provided evidence that lung adenocarcinoma patients with co-alterations of *EGFR*, *ROS1*, or *EML4-ALK* may benefit from TKIs treatment.

So far, there is little progress in digging the pathogenic mechanism of MPA or the treatment of this subtype by TKIs. Therefore, based on our findings, we will focus on elucidating the function of *ROS1* rearrangement and *EGFR* mutations in MPA by establishing the cell and animal models both in vitro and in vivo in the future. In addition, we will verify the efficacy of one targeted TKI or combined TKIs for MPA and provide the potential treatment strategy.

In summary, we report for the first time the relationship between the most common oncogenic mutations and pathological characteristics in Chinese lung adenocarcinoma patients. We discover the higher incidence of *ROS1* rearrangements and the coexistence of genetic alterations involving *EGFR*, *ROS1*, and *EML4-ALK* in MPA cases, indicating that targeting of *ROS1* combined with *EGFR* mutations may provide a novel therapeutic strategy for these patients. However, these results still should be supported by further studies with larger cases and in multi-centers.

## Data Availability

All data generated and analyzed during the present study are available from the corresponding author on reasonable request.

## Abbreviations

MPA: Micropapillary lung adenocarcinoma
LA: Lung adenocarcinoma without micropapillary component
EGFR: Epidermal growth factor receptor
ROS1: ROS proto-oncogene 1 receptor tyrosine kinase
ALK: Anaplastic lymphoma kinase
EML4-ALK: Echinoderm microtubule-associated protein-Like 4-anaplastic lymphoma kinase
KRAS: Kirsten rat sarcoma viral oncogene
HER2: Erb-B2 receptor tyrosine kinase 2
RET: Rearranged during transfection
MET: Mesenchymal-epithelial transition
FFPE: Formalin-fixed paraffin-embedded
TKI: Tyrosine kinase inhibitor
TNM: Tumor node metastasis
IASLC: International Association for the Study of Lung Cancer
ATS: American Thoracic Society
ERS: European Respiratory Society
ARMS: Amplification refractory mutation system
PCR: Polymerase chain reaction
IHC: Immunohistochemistry
NGS: Next generation sequencing
FISH: Fluorescence in situ hybridization
RECIST 1.1: Response Evaluation Criteria in Solid Tumors version 1.1
ORR: Objective response rate
CR: Complete response
PR: Partial response
SD: Stable disease
PD: Progressive disease
